# Nomogram prediction of the 70-gene signature (MammaPrint) binary and quartile categorized risk using medical history, imaging features and clinicopathological data among Chinese breast cancer patients

**DOI:** 10.1186/s12967-023-04523-7

**Published:** 2023-11-09

**Authors:** Bo Pan, Ying Xu, Ru Yao, Xi Cao, Xingtong Zhou, Zhixin Hao, Yanna Zhang, Changjun Wang, Songjie Shen, Yanwen Luo, Qingli Zhu, Xinyu Ren, Lingyan Kong, Yidong Zhou, Qiang Sun

**Affiliations:** 1 Department of Breast Surgery, Peking Union Medical College Hospital, Chinese Academy of Medical Sciences & Peking Union Medical College, Beijing, 100730 People’s Republic of China; 2 Department of Nuclear Medicine, Peking Union Medical College Hospital, Chinese Academy of Medical Sciences & Peking Union Medical College, Beijing, 100730 People’s Republic of China; 3 Department of Ultrasound, Peking Union Medical College Hospital, Chinese Academy of Medical Sciences & Peking Union Medical College, Beijing, 100730 People’s Republic of China; 4 Department of Pathology, Peking Union Medical College Hospital, Chinese Academy of Medical Sciences & Peking Union Medical College, Beijing, 100730 People’s Republic of China; 5 Department of Radiology, Peking Union Medical College Hospital, Chinese Academy of Medical Sciences & Peking Union Medical College, Beijing, 100730 People’s Republic of China

**Keywords:** Breast cancer, 70-gene signature (MammaPrint), Prognosis, Nomogram, Risk prediction

## Abstract

**Background:**

The 70-gene signature (70-GS, MammaPrint) test has been recommended by the main guidelines to evaluate prognosis and chemotherapy benefit of hormonal receptor positive human epidermal receptor 2 negative (HR + /Her2−) early breast cancer (BC). However, this expensive assay is not always accessible and affordable worldwide. Based on our previous study, we established nomogram models to predict the binary and quartile categorized risk of 70-GS.

**Methods:**

We retrospectively analyzed a consecutive cohort of 150 female patients with HR + /Her2− BC and eligible 70-GS test. Comparison of 40 parameters including the patients’ medical history risk factors, imaging features and clinicopathological characteristics was performed between patients with high risk (N = 62) and low risk (N = 88) of 70-GS test, whereas risk calculations from established models including Clinical Treatment Score Post-5 years (CTS5), Immunohistochemistry 3 (IHC3) and Nottingham Prognostic Index (NPI) were also compared between high vs low binary risk of 70-GS and among ultra-high (N = 12), high (N = 50), low (N = 65) and ultra-low (N = 23) quartile categorized risk of 70-GS. The data of 150 patients were randomly split by 4:1 ratio with training set of 120 patients and testing set 30 patients. Univariate analyses and multivariate logistic regression were performed to establish the two nomogram models to predict the the binary and quartile categorized risk of 70-GS.

**Results:**

Compared to 70-GS low-risk patients, the high-risk patients had significantly less cardiovascular co-morbidity (p = 0.034), more grade 3 BC (p = 0.006), lower progesterone receptor (PR) positive percentage (p = 0.007), more Ki67 high BC (≥ 20%, p < 0.001) and no significant differences in all the imaging parameters of ultrasound and mammogram. The IHC3 risk and the NPI calculated score significantly correlated with both the binary and quartile categorized 70-GS risk classifications (both p < 0.001). The area under curve (AUC) of receiver-operating curve (ROC) of nomogram for binary risk prediction were 0.826 (C-index 0.903, 0.799–1.000) for training and 0.737 (C-index 0.785, 0.700–0.870) for validation dataset respectively. The AUC of ROC of nomogram for quartile risk prediction was 0.870 (C-index 0.854, 0.746–0.962) for training and 0.592 (C-index 0.769, 0.703–0.835) for testing set. The prediction accuracy of the nomogram for quartile categorized risk groups were 55.0% (likelihood ratio tests, p < 0.001) and 53.3% (p = 0.04) for training and validation, which more than double the baseline probability of 25%.

**Conclusions:**

To our knowledge, we are the first to establish easy-to-use nomograms to predict the individualized binary (high vs low) and the quartile categorized (ultra-high, high, low and ultra-low) risk classification of 70-GS test with fair performance, which might provide information for treatment choice for those who have no access to the 70-GS testing.

**Supplementary Information:**

The online version contains supplementary material available at 10.1186/s12967-023-04523-7.

## Introduction

Breast cancer (BC) is the commonest malignancy worldwide and the leading cause of cancer death in Chinese women younger than 45 years [1–3]. In 2020, nearly 2,261,419 women were newly diagnosed breast cancer and 684,996 died from breast cancer, with a cumulative lifetime (age range: 0–74 years) risk of 5.20% [1, 4]. Hormonal receptor positive human epidermal receptor 2 negative (HR + /Her2−) comprises of approximately 60% of all BC [5], and multiple gene expression profiles are frequently used to evaluated the recurrence risk and benefit from adjuvant chemotherapy for HR + /Her2− early breast cancer (EBC) patients [6–8]. Currently, the 70-gene signature (70-GS, MammaPrint) test and 21-gene recurrence score (RS) have been validated in large prospective cohort, and recommended by the National Comprehensive Cancer Network (NCCN) guidelines as the mainstream of gene expression assays for HR + /Her2− EBCs [9].

Van’t Veer et al. analyzed treatment-naive EBC samples with DNA microarray analysis and found this 70-GS covering 7 pathways related to tumor proliferation, angiogenesis, invasion and migration which could predict risk of distance metastases [10]. In MINDACT trial, 46% of patients at clinical high risk (C-high) were assessed as genetic low risk (G-low) by MP 70-GS, and this group of patients could spare chemotherapy safely. A prospective multicenter study including 660 HR + /Her2− EBC patients showed that MP 70-GS changed half of the physician-intended recommendation of adjuvant chemotherapy [11]. Another study also demonstrated that use of the 70-GS changed patients’ inclination to receive adjuvant chemotherapy and facilitated decision-making [12]. Patients with 70-GS ultralow risk manifested good prognosis which was even distinctive from the 70-GS low risk, with 8-year breast cancer-specific survival (BCSS) rate of 99.6%, and distant metastasis-free interval (DMFI) rate of 97% [13] and might potentially be candidates for further de-escalation of treatment including the duration endocrine therapy [13, 14]. In the neoadjuvant setting, the adaptive randomized I-SPY2 trial and the observational prospective NBRST trial showed that the MP 70-GS high and ultrahigh risk could be associated with pathological complete response (pCR) rate and determine the chemo-sensitivity and long-term outcomes as predictive and prognostic biomarker [15, 16].

Previously we established immunohistochemistry 3 (IHC3) model based on the 21-gene RS and survival data to evaluate the personalized prognosis of HR + /Her2− EBCs and guide treatment choice [17]. We also combined the Clinical Treatment Score post-5 years (CTS5) model and 21-gene RS to develop a novel nomogram for prognosis prediction [18]. Study on BC patients among African-American females (AAF) who has unfavorable outcome compared to Caucasians showed that the 21-gene RS and 70-GS offered different prognostic information [19]. Another study revealed that 70-GS could provide useful information in addition to 21-gene assay resulting in changes of treatment decision in 33.6% of HR + /Her2− BC patients [20]. Given the expenses for the 70-GS assay, it is not always available and affordable worldwide, particularly in developing countries. There is little information about distribution of 70-GS risk among Chinese women and prediction models for 70-GS risk.

In this study, we planned to establish a nomogram model based on individualized medical history, imaging features and clinicopathological characteristics to predict the binary (high/low) and quartile categorized (ultrahigh, high, low, ultralow) risk of 70-GS test in HR + /Her2− EBCs among Chinese from a consecutive clinical cohort.

## Patients and methods

### Ethics statement

This retrospective study was approved by the Ethics Committee of the Peking Union Medical College Hospital (PUMCH), Chinese Academy of Medical Sciences.

### Patient population

**Fig. 1:**
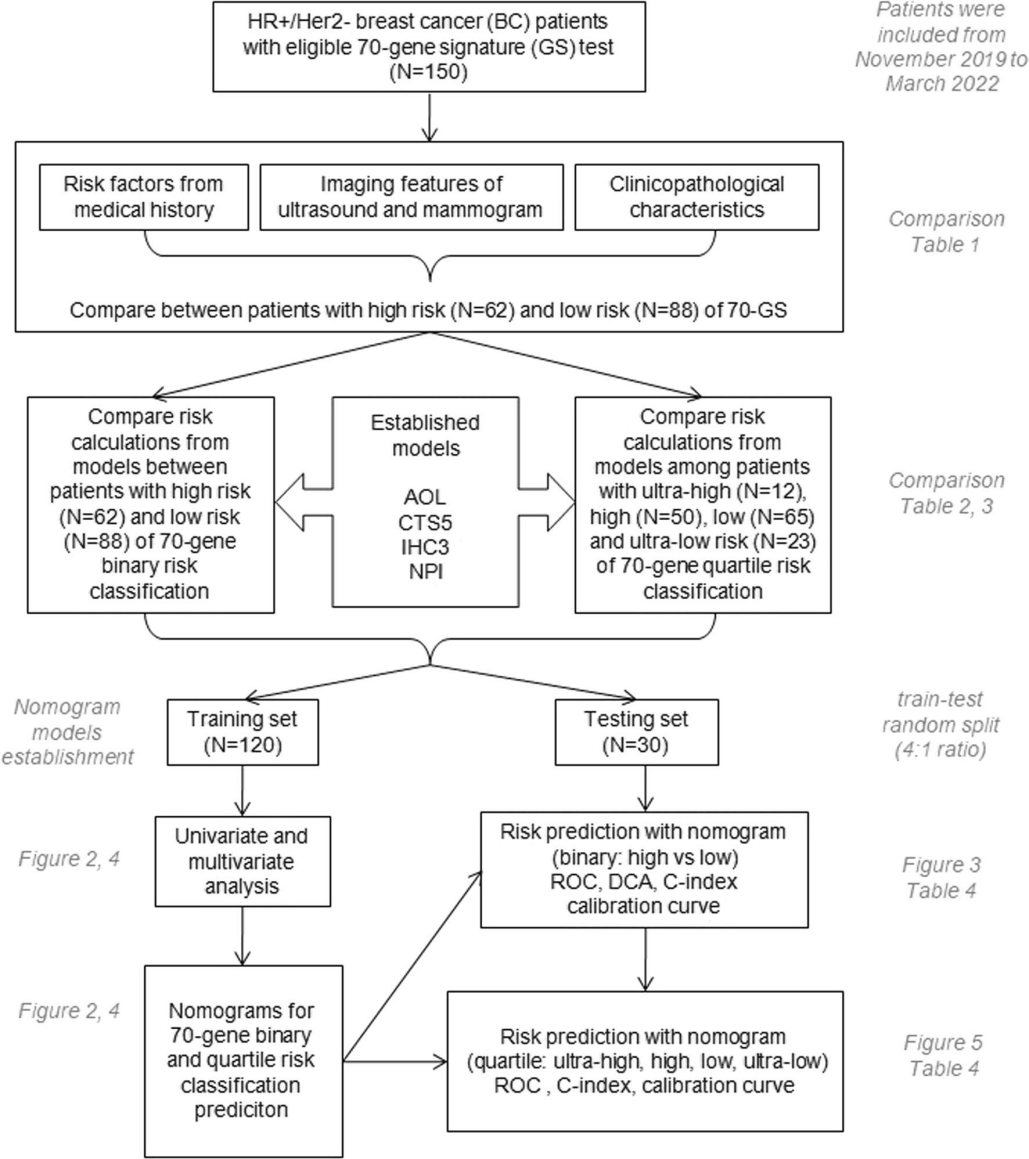
Flowchart of the study design with case number of each risk group of patients with eligible 70-gene signature test. The annotation and the number of tables and figures in accordance with the comparison and analysis results were italicized and in gray

There were 150 consecutive female patients diagnosed with HR + /Her2− breast cancer and received treatment in Dept. Breast Surgery, PUMCH from November 2019 to March 2022. The 70-GS (MammaPrint) test was performed by ZhenHe Genecast Biotechnology Ltd, sole and exclusive appointed partner of 70-GS assay in China by Agendia. Patients’ medical history, reports of ultrasound (US) and mammogram (MG) and clinicopathological characteristics were reviewed collected (Fig. 1).

### Comparison of medical history risk factors, imaging features and clinicopathological characteristics between high vs low risk of 70-GS test

Comparison of 40 parameters including the patients’ medical history risk factors, imaging features and clinicopathological characteristics was performed between patients with high risk (N = 62) and low risk (N = 88) of 70-GS test. Imaging features including MG density, micro-calcification cluster, nodule/mass and breast imaging reporting and data system (BI-RADS) category, US aspect ratio, boundary, morphology, hyperechoity, multicentricity/multifocality, blood flow, lymph node condition and BI-RADS category were extracted from imaging reports and coded for comparison (Fig. 1).

### Comparisons of risk calculations from established models among patients with different categories of 70-GS risk

Comparisons of risk calculations from established models including Adjuvant! Online (AOL) version 8.0 (Additional file 1: Fig. S1) [21], CTS5 [18, 22], IHC3 [17] and Nottingham prognostic index (NPI) [23] were performed between patients with high risk (N = 62) and low risk (N = 88) of 70-GS binary risk classification, as well as among patients with ultra-high (N = 12, defined as 70-GS score < − 0.569) [24], high (N = 50, 70-GS score − 0.569–0), low (N = 65, 70-GS score 0–0.355) and ultra-low risk (N = 23, 70-GS score > 0.355) of 70-gene quartile categorized risk classification [13].

### Establishment and validation of nomogram models to predict binary and quartile categorized risk of 70-GS

The data of 150 patients were randomly split by 4:1 ratio and the training set included data from 120 patients and testing set 30 patients. Univariate analyses and multivariate logistic regression were performed both based on binary 70-GS risk classification (high vs low risk), and based on quartile categorized risk classification (ultra-high, high, low and ultra-low risk). Two nomograms were established to predict the binary and quartile risk categories of 70-GS  (Fig. 1).

### Statistical analysis

The quantitative variables were compared with t-test, the categorical variables with chi-square tests. Univariate analysis was performed to identify variables associated with 70-GS risk. Multivariate logistic regression was used to develop the nomogram models. Risk predictors were selected using both stepwise regression analyses which based on Akaike information criterion (AIC), clinical importance and our previous study. Area under curve (AUC) of receiver operating characteristic (ROC) curves and C-index with 95% confidence interval (CI) was calculated to evaluate accuracy and discrimination of nomogram models. Calibration curve were used for visual inspection of calibration. The decision curve analysis (DCA) was used to reveal the potential clinical utility only for nomogram model of binary risk prediction. Statistical analyses were performed using R (4.0.3) software. All the statistical tests were two-sided, and statistical significance was defined as p value < 0.05.

## Results

### Comparison of medical history risk factors, imaging features and clinicopathological characteristics between 70-GS high vs low risk patients

Compared to 70-GS low-risk patients, the 70-GS high-risk patients had less cardiovascular co-morbidity (12.9% vs 27.3%, p = 0.034), more grade 3 BC (19.4% vs 4.5%, p = 0.006), lower progesterone receptor (PR) positive percentage (53.92 ± 36.49% vs 68.83 ± 30.43%, p = 0.007), more Ki67 high BC (≥ 20%, 87.1% vs 45.5%, p < 0.001) (Table 1). There were no significant differences in age, body mass index (BMI), childbirth, menarche, menopause, screen-detected BC, bilateral BC, all the included imaging parameters, TNM stage, multi-focality, lymphovascular invasion (LVI), estrogen receptor (ER) positivity and Her2 expression (Table 1).

**Table 1:** Comparison of medical history risk factors, imaging features and clinicopathological characteristics between Chinese patients with high versus low risk of 70-gene signature test

Clinicopathological and imaging characteristics	70-gene high risk = N = 62 (%)	70-gene low risk N = 88 (%)	P-value
70-gene score (Mean ± SD)	− 0.282 ± 0.234	0.226 ± 0.153	< 0.001
Medical history factors			
Age (Mean ± SD)	51.03 ± 9.67	52.27 ± 10.98	0.476
Age group			
< 40	8 (12.9)	9 (10.2)	0.705
40 ~ 49	21 (33.9)	33 (37.5)	
50 ~ 59	21 (33.9)	24 (27.3)	
≥ 60	12 (19.3)	22 (25.0)	
BMI (Mean ± SD)	23.57 ± 3.17	23.49 ± 3.11	0.879
Childbirth (Mean ± SD)	1.21 ± 0.70	1.16 ± 0.69	0.663
Age of menarche (Mean ± SD)	13.95 ± 1.77	13.47 ± 1.36	0.059
Age of menopause (Mean ± SD)	50.58 ± 2.99	50.34 ± 2.46	0.719*
Family history of breast, ovarian and pancreatic cancer			
No	58 (93.5)	76 (86.4)	0.189^
Yes	4 (6.5)	12 (13.6)	
Cardiovascular disease as co-morbidity			
No	54 (87.1)	64 (72.7)	0.034
Yes	8 (12.9)	24 (27.3)	
Screen-detected NPBC			
No	53 (85.5)	73 (83.0)	0.677
Yes	9 (14.5)	15 (17.0)	
Bilateral cancer			
No	60 (96.8)	85 (96.6)	0.945^
Yes	2 (3.2)	3 (3.4)	
Imaging features			
MG BI-RADS density			
1	5 (8.1)	9 (10.2)	0.385^#^
2	6 (9.7)	17 (19.3)	
3	34 (54.8)	42 (47.7)	
4	2 (3.2)	5 (5.7)	
Unknown	15 (17.0)	15 (24.2)	
MG micro-calcification cluster			
No	23 (37.1)	44 (50.0)	0.222^#^
Yes	24 (38.7)	29 (33.0)	
Unknown	15 (24.2)	15 (17.0)	
MG nodule/mass			
No	14 (22.6)	26 (29.5)	0.509^#^
Yes	33 (53.2)	47 (53.4)	
Unknown	15 (24.2)	15 (17.0)	
MG lesion BI-RADS			
2, 3 and 4a	12 (19.4)	26 (29.5)	0.274^#^
4b and 4c	23 (37.1)	25 (28.4)	
5 and 6	10 (16.1)	18 (20.5)	
Unknown	17 (27.4)	19 (21.6)	
US lesion BI-RADS			
2, 3 and 4a	4 (6.5)	6 (6.8)	0.340^#^
4b and 4c	35 (56.5)	41 (46.6)	
5 and 6	19 (30.6)	30 (34.1)	
Unknown	4 (6.5)	6 (6.8)	
US max. diameter of tumor (Mean ± SD, cm)	2.06 ± 0.73	2.10 ± 1.02	0.785^#^
US min. diameter of tumor (Mean ± SD, cm)	1.33 ± 0.46	1.24 ± 0.49	0.280^#^
US diameter Ratio (min/max) (Mean ± SD)	0.67 ± 0.15	0.64 ± 0.17	0.292^#^
US aspect ratio			
Normal	51 (82.3)	68 (77.3)	0.414^#^
Abnormal	7 (11.3)	14 (15.9)	
Unknown	4 (6.5)	6 (6.8)	
US boundary			
Clear	6 (9.7)	8 (9.1)	0.909^#^
Unclear	52 (83.9)	74 (84.1)	
Unknown	4 (6.5)	6 (6.8)	
US morphology			
Regular	3 (4.8)	4 (4.5)	0.941^#^^
Irregular	55 (88.7)	78 (88.6)	
Unknown	4 (6.5)	6 (6.8)	
US hyperechoicity			
No	28 (45.2)	37 (42.0)	0.712^#^
Yes	30 (48.4)	45 (51.1)	
Unknown	4 (6.5)	6 (6.8)	
US focality			
Unifocal	41 (66.1)	54 (61.4)	0.546^#^
Multifocal	17 (27.4)	28 (31.8)	
Unknown	4 (6.5)	6 (6.8)	
US blood flow			
Normal	28 (45.2)	45 (51.1)	0.441^#^
Abnormal	30 (48.4)	37 (42.0)	
Unknown	4 (6.5)	6 (6.8)	
US lymph node			
Normal	49 (79.0)	68 (77.3)	0.807^#^
Abnormal	9 (14.5)	14 (15.9)	
Unknown	4 (6.5)	6 (6.8)	
Clinicopathological characteristics			
Tumor histology			
IDC-NOS	56 (90.3)	83 (94.3)	0.355
Other	6 (9.7)	5 (5.7)	
pT			
T1	38 (61.3)	60 (68.2)	0.602^^^
T2	22 (35.5)	26 (29.5)	
T3	2 (3.2)	2 (2.3)	
Tumor volume (Mean ± SD, cm^3^)	3.68 ± 5.46	3.97 ± 11.28	0.849
pN			
N0	35 (56.5)	37 (42.10)	0.082
N1	27 (43.5)	51 (58.0)	
Number of positive nodes			
0	35 (56.5)	37 (42.0)	0.371^^^
1	21 (33.9)	37 (42.0)	
2	5 (8.1)	11 (12.5)	
3	1 (1.6)	3 (3.4)	
TNM stage			
I	35 (56.5)	37 (42.0)	0.082
II	27 (43.5)	51 (58.0)	
Histological grade			
G1	2 (3.2)	10 (11.4)	0.006
G2	48 (77.4)	74 (84.1)	
G3	12 (19.4)	4 (4.5)	
LVI			
No	53 (85.5)	77 (87.5)	0.721
Yes	9 (14.5)	11 (12.5)	
ER positivity (%) (Mean ± SD)	87.40 ± 11.20	87.70 ± 8.02	0.848
ER positive level			
Strong (3 +)	44 (71.0)	69 (78.4)	0.298
Mild/Moderate (1–2 +)	18 (29.0)	19 (21.6)	
PR positivity (%) (Mean ± SD)	53.92 ± 36.49	68.83 ± 30.43	0.007
PR positive level			
High (≥ 20%)	58 (93.5)	84 (95.5)	0.718^^^
Low (< 20%)	4 (6.5)	4 (4.5)	
PR positive level			
Strong (3 +)	37 (59.7)	64 (72.7)	0.199^^^
Moderate (2 +)	20 (32.3)	21 (23.9)	
Mild (1 +)	5 (8.1)	3 (3.4)	
Her2			
0	15 (24.2)	23 (26.1)	0.845
1 +	23 (37.1)	35 (39.8)	
2 +	24 (38.7)	30 (34.1)	
Her2 FISH ratio (Mean ± SD)	1.28 ± 0.38	1.22 ± 0.95	0.768^$^
Ki67			
High (≥ 20%)	54 (87.1)	40 (45.5)	**< 0.001**
Low (< 20%)	8 (12.9)	48 (54.5)	

*BC* breast cancer, *SD* standard deviation, *BMI* body mass index; *NPBC* non-palpable breast cancer, *MG* mammogram, *US*, ultrasound, *BI-RADS* breast imaging reporting and data system; *IDC-NOS* invasive ductal carcinoma not otherwise specified, *TNM* tumor-node-metastasis, *LVI* lymphovascular invasion, *ER* estrogen receptor, *PR* progesterone receptor, *FISH* fluorescence in situ hybridization

^*^The comparison was performed without the perimenopausal patients

^#^The comparison was performed without unknown cases

^The comparison was performed by fisher test

^$^The comparison was performed only for Her2 (2 +) patients

### Comparisons of risk calculations from established models among patients with different categories of 70-GS risk

There was no significant difference in CTS5 score and in percentage of high-risk patients evaluated by AOL, CTS5 and NPI models between 70-GS binary risk groups of patients (Table 2) or in quartile categorized risk classification of patients (Table 3). There were more high-risk patients evaluated by IHC3 model in the 70-GS high-risk group (43.5% vs 11.4%, p < 0.001) (Table 2), and the percentage of IHC3 risk decreased accordingly among 70-GS ultra-high, high, low and ultra-low risk subgroups of patients (83.3%, 34.0%, 15.4% and 0.0%, p < 0.001) (Table 3). The NPI score also decreased accordingly with the 70-GS risk (Table 3).

**Table 2:** Comparison of risk calculated from established models between Chinese patients with high versus low risk based on binary risk classification of 70-gene signature test

Risk calculated from established models	70-gene high risk N = 62 (%)	70-gene low risk N = 88(%)	P-value
AOL			
High	46 (74.2)	61 (69.3)	0.516
Low	16 (25.8)	27 (30.7)	
CTS5			
Score (Mean ± SD)	3.29 ± 0.58	3.14 ± 0.64	0.137
CTS5			
High	8 (12.9)	12 (13.6)	0.035
Intermediate	33 (53.2)	29 (33.0)	
Low	21 (33.9)	47 (53.4)	
IHC3			
High	27 (43.5)	10 (11.4)	< 0.001
Low	35 (56.5)	78 (88.6)	
NPI			
Score (Mean ± SD)	4.58 ± 0.49	4.30 ± 0.52	0.001
NPI			
Poor	0 (0.0)	1 (1.1)	0.348^^^
Moderate	48 (77.4)	60 (68.2)	
Good	14 (22.6)	27 (30.7)	

*AOL* Adjuvant! Online, *CTS5* Clinical treatment Score post–5 years, IHC3, immunohistochemistry 3, *NPI* Nottingham prognostic index

^The comparison was performed by fisher test

**Table 3:** Comparison of risk predicted from established models among Chinese patients with ultra-high, high, low and ultra-low risk based on quartile risk classification of 70-gene signature test

Risk calculated from established models	70-gene ultrahigh risk N = 12 (%) 4	70-gene high risk N = 50 (%) 3	70-gene low risk N = 65 (%) 2	70-gene ultralow risk N = 23 (%) 1	P-value
AOL					0.861^&^
High (1)	9 (75.0)	37 (74.0)	44 (67.7)	17 (73.9)	
Low (0)	3 (25.0)	13 (26.0)	21 (32.3)	6 (26.1)	
CTS5					0.162
Score (Mean ± SD)	3.34 ± 0.41	3.28 ± 0.62	3.14 ± 0.63	3.13 ± 0.66	
CTS5					0.250^^^
High (2)	1 (8.3)	7 (14.0)	9 (13.8)	3 (13.0)	
Intermediate (1)	8 (66.7)	25 (50.0)	22 (33.8)	7 (30.4)	
Low (0)	3 (25.0)	18 (36.0)	34 (52.3)	13 (56.5)	
IHC3					< 0.001^^^
High (1)	10 (83.3)	17 (34.0)	10 (15.4)	0 (0.0)	
Low (0)	2 (16.7)	33 (66.0)	55 (84.6)	23 (100.0)	
NPI					< 0.001
Score (Mean ± SD)	4.83 ± 0.58	4.52 ± 0.45	4.34 ± 0.46	4.18 ± 0.65	
NPI					0.768^^^
Poor (2)	0 (0.0)	1 (1.5)	0 (0.0)	0 (0.0)	
Moderate (1)	9 (75.0)	39 (78.0)	43 (66.2)	17 (73.9)	
Good (0)	3 (25.0)	11 (22.0)	21 (32.3)	6 (26.1)	

*AOL* Adjuvant! Online, *CTS5* Clinical treatment Score post–5 years;* IHC3* immunohistochemistry 3,* NPI* Nottingham prognostic index

^&^The comparison was performed by Chi square test of continuous correction

^The comparison was performed by fisher test

### Establishment and validation of nomogram models to predict binary and quartile categorized risk of 70-GS

**Fig. 2:**
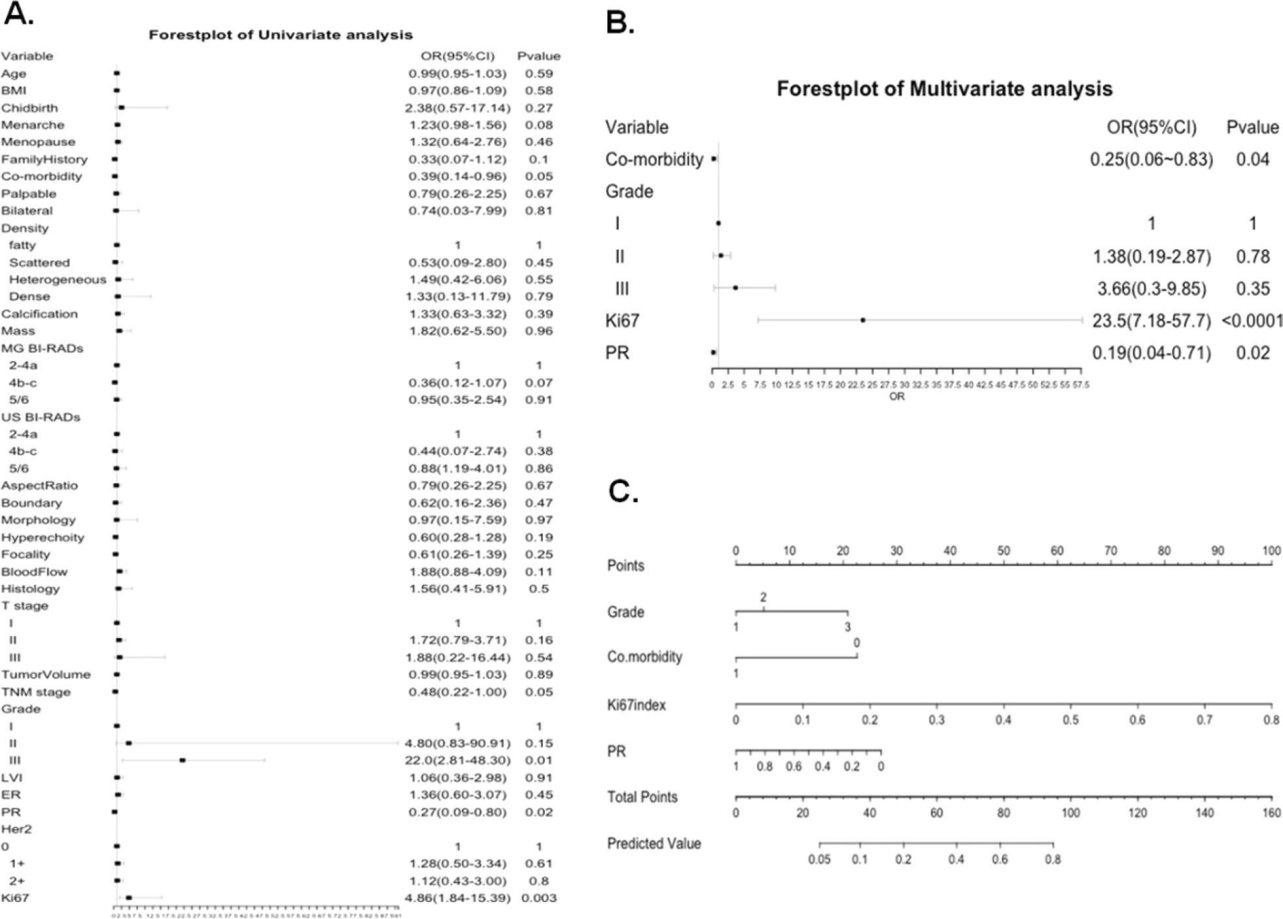
Forest plots of univariate (**A**) and multivariate (**B**) analyses of logistic regression showing the risk factors included and the according nomogram model (**C**) based on binary risk classification (high vs low risk) of 70-gene signature test

The risk factors identified by the univariate analyses and multivariate logistic regression included cardiovascular co-morbidity, histological grade, PR positive percentage and Ki67 index for both nomograms (Figs. 2, 4). The points for each factor were marked on the scale and the total points for each individual could indicate the possibility of high risk (Fig. 2) based on binary risk classification (high vs low) as well as the possibility of ultra-high, high or low risk (Fig. 4) based on quartile categorized risk classification (ultra-high, high, low and ultra-low risk) of 70-GS test.

**Fig. 3:**
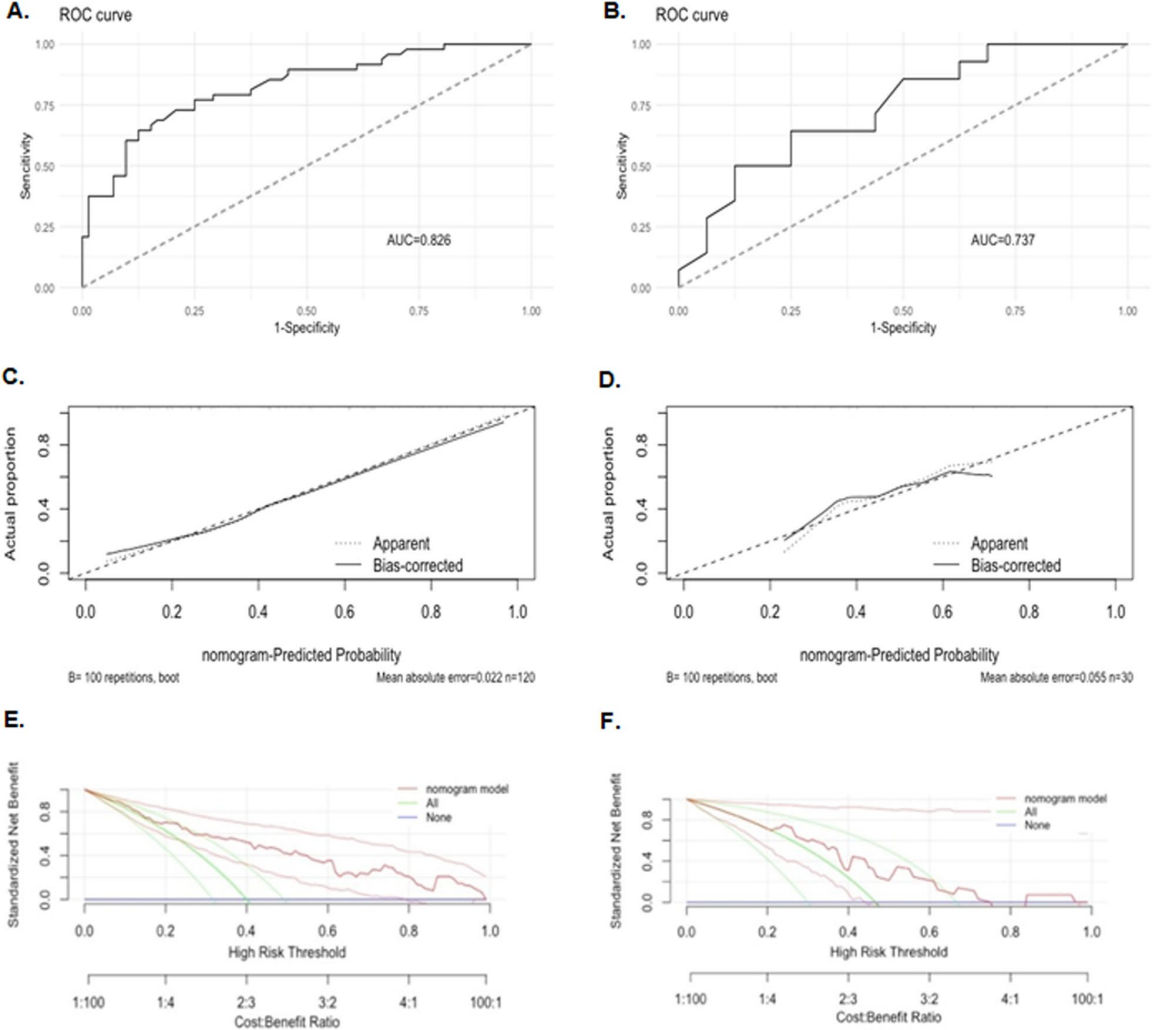
Receiver operating characteristic (ROC) curve (**A**), calibration curve (**C**) and decision curve analysis (DCA) (**E**) of the training set (N = 120) as well as the ROC curve (**B**), calibration curve (**D**) and DCA analysis (**F**) of the testing set (N = 30) from the established nomogram model (Fig. 2C) based on binary risk classification (high vs low risk) of 70-gene signature test

**Fig. 4:**
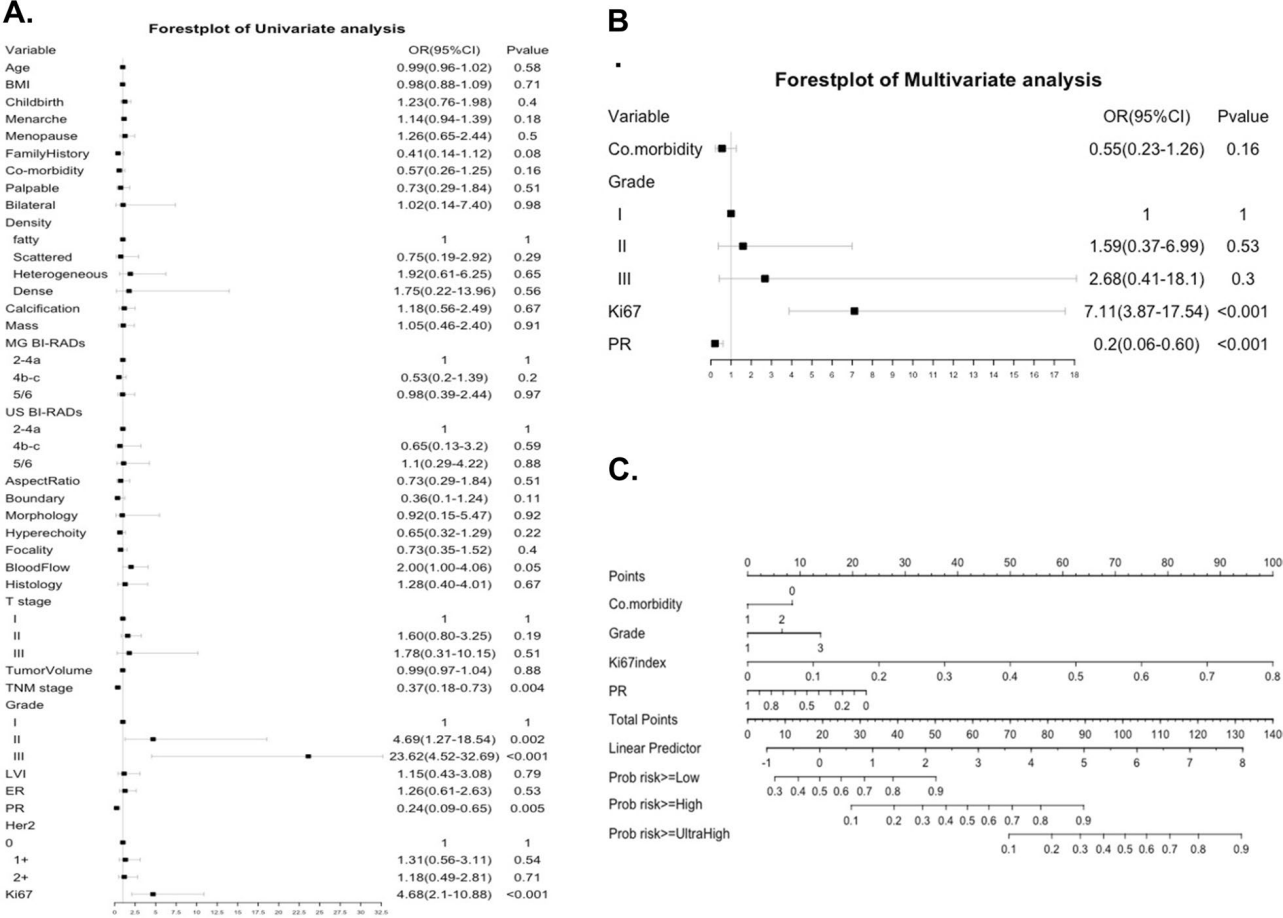
Forest plots of univariate (**A**) and multivariate (**B**) analyses of logistic regression showing the risk factors included and the according nomogram model (**C**) based on quartile categorized risk classification (ultra-high, high, low and ultra-low risk) of 70-gene signature test

**Fig. 5:**
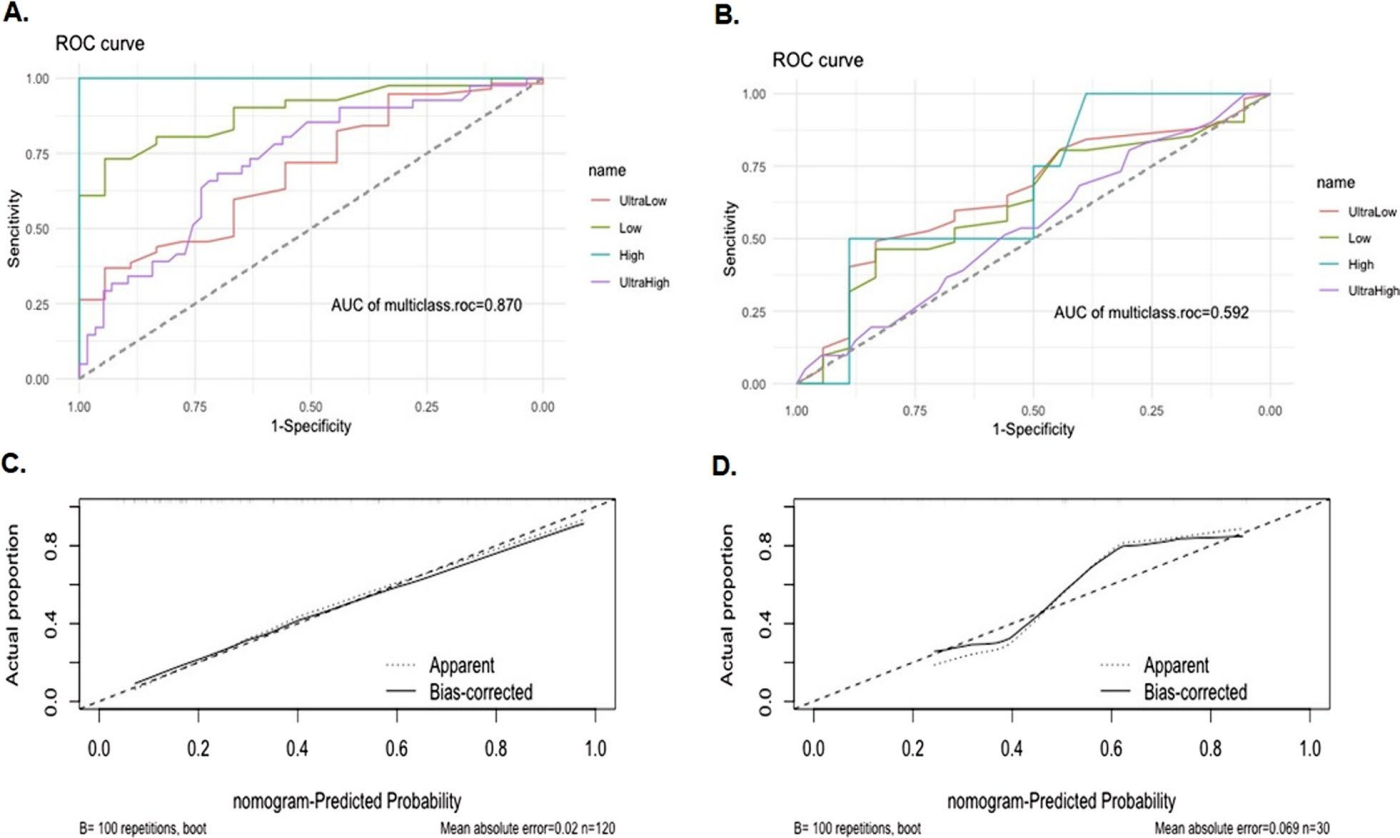
Receiver operating characteristic (ROC) curve (**A**) and calibration curve (**C**) of the training set (N = 120) and counterparts (**B**, **D**) of the testing set (N = 30) from the established nomogram model (Fig. 4C) based on quartile categorized risk classification (ultra-high, high, low and ultra-low risk) of 70-gene signature test

The calibration plots indicated the predicted 70-GS risk generated by the nomograms had a good consistency with the original 70-GS risk tested (Figs. 3, 5). The DCA indicated that when the threshold for predicted probability of high risk (binary risk classification) was within the range of 0.2–0.8, the nomogram model would add more net benefit than “all or none” strategy. The AUC of ROC curves of the nomogram for binary high risk prediction were 0.826 (C-index 0.903, 95%CI 0.799–1.000) for training and 0.737 (C-index 0.785, 95%CI 0.700–0.870) for validation dataset respectively (Table 4, Figs. 2, 3). The AUC of ROC of nomogram for quartile risk prediction was 0.870 (C-index 0.854, 95%CI 0.746–0.962) for training and 0.592 (C-index 0.769, 95%CI 0.703–0.835) for testing set (Table 4, Figs. 4, 5). The prediction accuracy of the nomogram for quartile categorized risk groups were 55.0% (likelihood ratio tests, p < 0.001) and 53.3% (p = 0.04) for training and validation, which more than double the baseline probability of 25%. The AIC was 128.54 (training) and 46.16 (testing) for binary risk nomogram and 247.07 (training) and 73.85 (testing) for quartile risk nomogram.

**Table 4:** Parameters include area under curve (AUC) and C-index to evaluate the accuracy and discrimination of binary and quartile categorized nomogram models

Nomogram models\Parameters to evaluate the nomograms	Binary		Quartile	
	Training set (N = 120)	Testing set (N = 30)	Training set (N = 120)	Testing set (N = 30)
AUC	0.826	0.737	0.870	0.592
C-index (95% CI)	0.903 (0.799–1.000)	0.785 (0.700–0.870)	0.854 (0.746–0.962)	0.769 (0.703–0.835)

*AUC* area under curve, *CI* confidence interval

## Discussion

Multi-gene assays are used worldwide for prognostic evaluation and predictive information beyond histological parameters for escalation and de-escalation of individualized treatment of HR + /Her2− BC patients. The 21-gene recurrence score (RS) has been validated as quantifying the likelihood of distant recurrence in tamoxifen treated patients with HR + /Her2− BC [25] as well as the potential benefit from adjuvant chemotherapy according to the TAILORx and RxPONDER trial [7, 26]. Study showed that the prognostic and predictive value of the 21-gene RS was consistent in different countries [27], however, there might be difference in the 21-gene assay performance across racial groups with better performance among white compared with African American and Hispanic individuals. [28]

The 21-gene RS could be predicted by nomograms and machine-learning models based on clinicopathological parameters [29–32]. The 70-GS further stratifies HR + /Her2− BC patients with clinically high-risk according to AOL model into binary (high vs low) or quartile categorized (ultra-high, high, low and ultra-low) risk classifications [6, 33]. Study revealed that the 70-GS could provide additional information regarding patients classified as intermediate risk by the 21-gene assay resulting in changes of treatment decision in 33.6% of HR + /Her2− BC patients [20]. Chemotherapy was added or withheld by the treating physician with more confidence based on the results of the 70-GS test [20]. Given the considerable expenses of the 70-GS assay (approximately 19,800 RMB Yuan, USD 2828$), there was also endeavor to establish deep learning model to predict the binary 70-GS risk from microscopic pathological whole slide images (WSIs) [34]. However, easy-to-use prediction models for 70-GS risk are still lacking.

To our knowledge our study was the first to establish user-friendly nomograms based on imaging and clincopathological parameters to predict the 70-GS risk both in binary (high vs low) and quartile categorized (ultra-high, high, low and ultra-low) risk groups among Chinese population. It might reduce the cost of tumor genetic testing and address optimal management of early breast cancer patients. Moreover, it was in a format that can be translated to patients that would simplify decision-making on their part. Risk factor indicating hormonal conditions such as childbirth, menarche and menopause were not associated with 70-GS risk because all the patients’ BCs were already hormonal receptor positive. However, the co-morbidity of cardio-vascular disease provided additional information of the patient’s general condition and was associated with a lower 70-GS risk (Table 1, Figs. 2, 4). Interestingly, there were no significant differences in all the included imaging features of ultrasound and mammogram between 70-GS high vs low risk patients (Table 1). Not surprisingly, the most ‘classic’ clincopathological parameters associated with 70-GS risk were still histological grade, PR and Ki-67 index (Table 1, Figs. 2, 4), which was in accordance with studies building nomogram predicting 21-gene RS [17, 29, 30].

Majority (71.3%) of patients were clinical high risk according to AOL model. However, study showed that AOL model over-estimated survival of Asian BC patients [35], which might explain why there were 43 (28.7%) patients with AOL low risk and were still judged by their physician as ‘clinical’ high risk and received 70-GS test (Table 2). The CTS5 risk model was developed based on the data from the ATAC (Arimidex, Tamoxifen, Alone or in Combination) trial and the BIG (Breast International Group) 1–98 trial to estimate risk of late distant recurrence with parameters including age, tumor size, grade and lymph node [22]. Our previous study developed a nomogram combining both CTS5 and 21-gene RS could improve the evaluation of HR + /Her2− BC patients [18]. The NPI is used to determine prognosis for invasive BC of all subtypes which combines nodal status, tumor size and histological grade, stratifies patients with breast cancer into good, moderate, and poor prognostic groups with validation in large cohort [23, 36, 37]. The IHC3 model was developed in our previous study with parameters including Ki-67 index, PR positive percentage, tumor size and grade and it improved the evaluation of prognosis of HR + /Her2 BC patients compared to 21-gene RS [17]. Notably, the IHC3 risk and the NPI calculated score instead of the NPI risk group significantly correlated with both the binary (high vs low) and quartile categorized (ultra-high, high, low and ultra-low) risk classifications (both p < 0.001, Tables 2, 3). Thus, although the IHC3 model was developed to evaluate the lymph-node negative BC patients, it might also be used to evaluate patient with 1–3 positive nodes combined with the NPI score. For example, if a HR + /Her2 BC patient was evaluated as AOL clinical high-risk and IHC3 low-risk with a NPI score < 4.0, she might potentially spare or de-escalate chemotherapy (Table 3). Furthermore, if a HR + /Her2 BC patient was judged by both of our 70-GS nomogram as low-risk then she might also potentially spare or de-escalate chemotherapy (Figs. 2, 4).

The main reason for constructing a quartile risk model after the establishment and evaluation of the binary risk model is to improve the discrimination and recognition of those patients who were ultra-low risk (70-GS score 0.355–1) or ultra-high risk (70-GS score − 1 ~ − 0.569), who showed clinical prognostic significance and were justified for personalized treatment [13–16]. Although ROC curve is usually for accuracy evaluation of models predicting binary/dichotomic results [38], we managed to calculate ROC curves both for training and testing set for nomogram model predicting quartile risk (Fig. 3, Table 4). However, the DCA analysis is for evaluating alternative diagnostic and prognostic strategies only for models predicting binary/dichotomic results [39]. The threshold probability used in DCA analysis is used to determine both whether a patient is defined as test-positive or negative and to model the clinical consequences of true and false positives using a clinical net benefit function [40]. Therefore we did not perform DCA analysis for models predicting categorized/multiple classification.

Our study also has several limitations. First, it was a retrospective study with limited sample size, and the testing set is small with only 30 patients, which limited interpretation of the results and also made it difficult for establishment of prediction model with artificial intelligence. Second, the survival data was still unavailable in the currently study, so the prediction model could only be validated with 70-GS risk categories without the actual follow-up outcome. Therefore, our next-step research would be modification on the nomogram models with increased sample size and survival outcome. Third, the imaging features were extracted from the text from the reports of ultrasound and mammogram and there were no actual images analyzed by deep learning and integrated into prediction model. Fourth, the treatment information was not included in the currently study, yet different treatment may affect the prognosis.

## Conclusion

We established easy-to-use nomogram models to predict the individualized binary (high vs low) and the quartile categorized (ultra-high, high, low and ultra-low) risk classification of 70-GS test with acceptable performance, which could guide treatment decision making for those who have no access to the 70-GS testing.

### Supplementary Information

**Additional file 1: Fig. S1.** The Adjuvant! Online (AOL) version 8.0 risk model used in the MINDACT trial as well as in this study.

## Data Availability

The datasets analysed during the current study are available from the corresponding author on reasonable request.
