# Supplementary material for: Nomogram prediction of the 70-gene signature (MammaPrint) binary and quartile categorized risk using medical history, imaging features and clinicopathological data among Chinese breast cancer patients
Source: J Transl Med. 2023 Nov 9;21:798. doi: 10.1186/s12967-023-04523-7 (PMC10637017; doi:10.1186/s12967-023-04523-7)
Supplement: Supplementary file 1 — Additional file 1: Fig. S1. The Adjuvant! Online (AOL) version 8.0 risk model used in the MINDACT trial as well as in this study. [file 12967_2023_4523_MOESM1_ESM.docx]

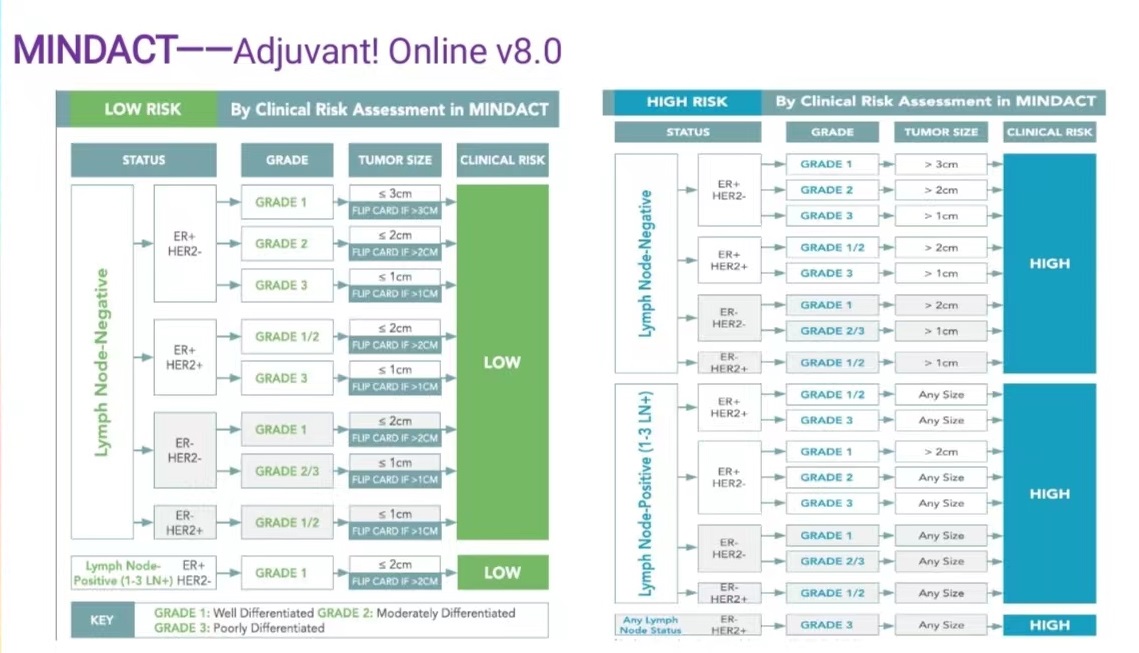


**Supplementary Figure 1.**The Adjuvant! Online (AOL) version 8.0 risk model used in the MINDACT trial as well as in this study.
